# Italian Society of Anesthesia, Analgesia, Resuscitation, and Intensive Care expert consensus statement on the use of lung ultrasound in critically ill patients with coronavirus disease 2019 (ITACO)

**DOI:** 10.1186/s44158-021-00015-6

**Published:** 2021-11-24

**Authors:** Luigi Vetrugno, Francesco Mojoli, Andrea Cortegiani, Elena Giovanna Bignami, Mariachiara Ippolito, Daniele Orso, Francesco Corradi, Gianmaria Cammarota, Silvia Mongodi, Enrico Boero, Carmine Iacovazzo, Maria Vargas, Daniele Poole, Daniele Guerino Biasucci, Paolo Persona, Tiziana Bove, Lorenzo Ball, Davide Chiumello, Francesco Forfori, Edoardo de Robertis, Paolo Pelosi, Paolo Navalesi, Antonino Giarratano, Flavia Petrini

**Affiliations:** 1 Department of Medicine, University of Udine, Via Colugna n 50, 33100 Udine, Italy; 2 University-Hospital of Friuli Centrale, ASU FC, Udine, Italy; 3 Department of Clinical-Surgical, Diagnostic, and Pediatric Sciences, Unit of Anesthesia and Intensive Care, University of Pavia, Pavia, Italy; 4 Department of Surgical, Oncological and Oral Science (Di.Chir.On.S), University of Palermo, Palermo, Italy; 5 Department of Anesthesia Intensive Care and Emergency, Policlinico Paolo Giaccone, Palermo, Italy; 6 Anesthesiology, Critical Care and Pain Medicine Division, Department of Medicine and Surgery, University of Parma, Parma, Italy; 7 Department of Surgical, Medical and Molecular Pathology and Critical Care Medicine, University of Pisa, Pisa, Italy; 8 Department of Anesthesia and Intensive Care, “Ente Ospedaliero Ospedali Galliera”, Genova, Italy; 9 Department of Medicine and Surgery, University of Perugia, Perugia, Italy; 10 Anesthesia and Intensive Care Unit, San Giovanni Bosco Hospital, Turin, Italy; 11 Department of Neurosciences, Reproductive and Odontostomatological Sciences, University of Naples “Federico II”, Naples, Italy; 12 Anesthesia and Intensive Care Operative Unit, S. Martino Hospital, Belluno, Italy; 13 Department of Anesthesia and Intensive Care, Fondazione Policlinico Universitario “A. Gemelli”, Rome, Italy; 14 UOC Anesthesia and Intensive Care Unit, University Hospital of Padua, Padua, Italy; 15 Department of Surgical Sciences and Integrated Diagnostics (DISC), University of Genoa, Genoa, Italy; 16 Anesthesia and Critical Care, San Martino Policlinico Hospital, IRCCS for Oncology and Neurosciences, Genoa, Italy; 17 Department of Anesthesia and Intensive Care, ASST Santi Paolo e Carlo, San Paolo University Hospital, Milan, Italy; 18 Italian Society of Anesthesia, Analgesia, Resuscitation, and Intensive Care (SIAARTI), Rome, Italy

**Keywords:** Consensus, Coronavirus disease 2019, Intensive care, Lung ultrasound

## Abstract

**Background:**

To produce statements based on the available evidence and an expert consensus (as members of the Lung Ultrasound Working Group of the Italian Society of Analgesia, Anesthesia, Resuscitation, and Intensive Care, SIAARTI) on the use of lung ultrasound for the management of patients with COVID-19 admitted to the intensive care unit.

**Methods:**

A modified Delphi method was applied by a panel of anesthesiologists and intensive care physicians expert in the use of lung ultrasound in COVID-19 intensive critically ill patients to reach a consensus on ten clinical questions concerning the role of lung ultrasound in the following: COVID-19 diagnosis and monitoring (with and without invasive mechanical ventilation), positive end expiratory pressure titration, the use of prone position, the early diagnosis of pneumothorax- or ventilator-associated pneumonia, the process of weaning from invasive mechanical ventilation, and the need for radiologic chest imaging.

**Results:**

A total of 20 statements were produced by the panel. Agreement was reached on 18 out of 20 statements (scoring 7–9; “appropriate”) in the first round of voting, while 2 statements required a second round for agreement to be reached. At the end of the two Delphi rounds, the median score for the 20 statements was 8.5 [IQR 8.9], and the agreement percentage was 100%.

**Conclusion:**

The Lung Ultrasound Working Group of the Italian Society of Analgesia, Anesthesia, Resuscitation, and Intensive Care produced 20 consensus statements on the use of lung ultrasound in COVID-19 patients admitted to the ICU. This expert consensus strongly suggests integrating lung ultrasound findings in the clinical management of critically ill COVID-19 patients.

**Supplementary Information:**

The online version contains supplementary material available at 10.1186/s44158-021-00015-6.

## Introduction

The use of lung ultrasound (LUS) in the intensive care setting has increased during the coronavirus disease 2019 (COVID-19) pandemic. It is being employed as a diagnostic and monitoring tool in patients with COVID-19-related pneumonia—a condition which in some cases evolves into acute respiratory distress syndrome (ARDS) [1]. Lung ultrasound presents many advantages over other imaging techniques: it is readily available at the bedside, thus avoiding the need to transport patients to the radiology department, and it is radiation-free and highly repeatable, making it suitable for lung monitoring purposes [2]. Lung damage in COVID-19 pneumonia is mainly localized to the peripheral regions of the lungs, thus easily accessible to ultrasound [3]. The sensitivity and negative predictive value of LUS for COVID-19 pneumonia are both higher compared with those for chest X-ray [4]. Moreover, many studies show a close correlation between LUS and computed tomography (CT) scan findings [5]. Given the prolonged need for mechanical ventilation in COVID-19 and long intensive care unit (ICU) stay, repeated lung assessments are usually required. CT remains the reference imaging technique for lung assessment, but it is unsuitable as a monitoring tool due to its use of ionizing radiation. It also necessitates patient contact with healthcare providers outside the ICU, increasing the opportunity for this highly infectious disease to spread. The quantitative evaluation of lung disease by means of the LUS score provides a reliable method for assessing lung aeration in both ARDS and COVID-19, and may further help in monitoring lung recovery and in the daily optimization of ventilation strategies (i.e., positive end-expiration pressure [PEEP] titration, and the use of prone positioning) [6]. Finally, LUS permits the early bedside detection of complications, such as pneumothorax [7] and ventilator-associated pneumonia [8]. As a consequence, LUS has earned a leading position in the management of COVID-19 patients, being a reliable, time-sparing, and easy-to-learn alternative to traditional imaging techniques [9, 10]. However, the recent literature is mainly focused on its applications within the Emergency Department [11]. Although Canadian Internal Medicine Ultrasound (CIMUS) experts recently established their recommendations for medical inpatients with COVID-19 [12], consensus guidelines dedicated to COVID-19 ICU patients and officially acknowledged by a national intensive care scientific society are lacking. To fill this gap, we aimed to produce an expert consensus on the bedside use of LUS in critically ill COVID-19 patients by a national panel of anesthesiology and intensive care physicians.

## Methods

### Consensus process design

This project was conducted according to a modified Delphi method to reach consensus on key aspects of the use of LUS in critically ill patients with COVID-19. Discussions were based on the available scientific evidence as well as the panel of experts’ own clinical experience. The experts were selected by the project coordinators (LV and FM) based not only on their clinical and scientific interest in the topic [13] but also the opinion of intensivists who are not experts in LUS but who understand the context of critically ill COVID patients, and the potential role of lung ultrasound was invited. After an initial (online) kick-off meeting between the coordinators, the panelists, the methodologists (AC and DP), and the evidence review team (MI and DO), the project coordinators proposed a list of the most relevant clinical questions to the whole panel, which was then asked to submit a blind boolean vote (“agree/disagree on the relevance”) as well as comments and proposals for their modification. In response, the coordinators made the appropriate changes to the clinical questions, which were finally approved by the whole panel through a second round of voting. The coordinators then assigned the work on each clinical question to a designated group of experts, each of which was led by a designated group head (PP, PN, EB, TB, and SM). The list of clinical questions and the final consensus-based statements is shown in Table 1.

**Table 1:** List of clinical questions and final consensus-based statements

10-clinical questions	20-consensus-based statements
A) Does LUS have a role to play in the diagnosis of COVID-19?	**Statement 1:** LUS should be integrated in the clinical workup to diagnose COVID-19 pneumonia. **Statement 2:** In patients with high clinical suspicion of COVID-19 and LUS findings compatible with ultrasonographic interstitial pneumonia, a negative nasal/oropharyngeal RT-PCR should not be used alone to exclude COVID-19. **Statement 3:** LUS should not be used alone to rule out SARS-CoV-2 pneumonia in suspected COVID-19.
B) Can LUS help in the early assessment of COVID-19 severity in the Emergency Department and/or at ICU admission?	**Statement 4**: Sonographic multifocal and bilateral pleural and lung abnormalities, a high overall LUS score, and/or a high score in the gravity dependent areas can be used for the early assessment of COVID-19 severity in the Emergency Department and at ICU admission since all correlate with worsening patient outcomes – intended as the need for ICU admission, NIV, intubation, and a higher mortality rate.
C) Can LUS be used as a lung monitoring tool in COVID-19 patients undergoing non-invasive respiratory support (HFNC, CPAP, or NIV)?	**Statement 5:** In COVID-19 patients undergoing non-invasive respiratory support (HFNC, CPAP or NIV), LUS integrated with clinical and physiological parameters may contribute to predict non-invasive respiratory support outcome and early detect complications.
D) Can LUS be used as a lung monitoring tool in COVID-19 patients undergoing invasive mechanical ventilation?	**Statement 6:** LUS should be integrated into the multimodal assessment of disease progression and the response to treatments in mechanically ventilated COVID-19 patients. **Statement 7:** LUS should be integrated into the clinical decision-making process and monitoring of procedures, such as fibrobronchoscopy, in mechanically ventilated COVID-19 patients. **Statement 8:** LUS should be integrated into the clinical decision-making process and monitoring of treatments, such as antibiotics, in mechanically ventilated COVID-19 patients. **Statement 9:** LUS should be integrated into the clinical decision-making process and monitoring of fluid removal in mechanically ventilated COVID-19 patients.
E) Can LUS guide the titration of positive end expiratory pressure in severe COVID-19 patients receiving invasive mechanical ventilation?	**Statement 10:** LUS should be considered as an additional tool for PEEP titration in COVID-19.
F) Can LUS guide the use of prone positioning in patients with severe COVID-19?	**Statement 11:** LUS should be integrated into the assessment of patients considered potential responders to prone positioning, according to the focal and non-focal pattern of lung aeration loss. **Statement 12:** LUS can be used to monitor variations in lung aeration during prone positioning.
G) Can LUS help early diagnosis of pneumothorax in severe COVID-19 patients undergoing invasive mechanical ventilation?	**Statement 13:** LUS should be used in COVID-19 patients, in line with its clinical appropriateness as ascertained by studies in non-COVID-19 patients.
H) Can LUS help the early diagnosis of ventilator-associated pneumonia in severe COVID-19 patients undergoing invasive mechanical ventilation?	**Statement 14:** LUS should be used in COVID-19 patients, in line with its clinical appropriateness as ascertained by studies in non-COVID-19 patients.
I) Can LUS help the process of weaning severe COVID-19 patients from invasive mechanical ventilation?	**Statement 15:** We are unable to create any statement on the use of LUS in the assessment of patient readiness to sustain a spontaneous breathing trial (SBT). No study has evaluated the potential of LUS for this purpose in COVID-19 patients, and data in non-COVID-19 patients are conflicting. **Statement 16:** In association with standard clinical and physiological indexes, LUS can improve the prediction of SBT outcome in COVID-19 patients. **Statement 17:** In association with standard clinical and physiological indexes, LUS can improve the prediction of extubation failure in COVID-19 patients.
L) Can LUS decrease the need for radiologic chest imaging in severe COVID-19 patients?	**Statement 18:** In patients with known COVID-19 and severe symptoms, LUS should be performed instead of radiologic chest imaging as the first-line imaging test to monitor disease progression. **Statement 19:** LUS findings suggestive of pneumonia can render additional imaging techniques unnecessary, especially when the likelihood of an alternative diagnosis is low. **Statement 20:** In COVID-19 patients with severe symptoms, a negative LUS exam should prompt an additional radiologic chest imaging work-up.

### Systematic review of available evidence

A systematic review of MEDLINE, PubMed, EMBASE, and pre-print depositories (medRxiv and bioRxiv) was performed by the evidence review team with the added input of AC. The full search strategy can be found in Additional file 1. Inclusion and exclusion criteria and the PRISMA flow diagram (Fig. S1) of the inclusion/exclusion process can be found in Additional file 2 with the results of the first round of voting (Fig. S2). The full output of the search and the selection of potentially relevant articles (selected by MI and DO, according to the PICO questions and criteria described in Table S1, Additional file 2) can be found in Additional file 3. The last literature search was completed on 16 January 2021. Other sources (i.e., reference lists from relevant articles [i.e., the snowballing method] and online journal issues) were surveyed after the last literature search and up until the end of the first round of voting so that any further relevant articles could be identified and included. The output of the search together with the full texts of all relevant articles was then sent to all panelists. Following the evaluation of the evidence, each panelist produced their statement and rationale in response to each of the given questions.

### The Delphi rounds, consensus meeting

Two rounds of voting were held between March and June 2021. The respondents were blinded to each other’s responses. In the first round, expert panelists responded to the online questionnaire and were offered the possibility of adding their opinions using an open text box. The research assistance team (CC, see the “Acknowledgements” section), with the input of the methodologist (AC), assessed and presented the results from round 1 in the form of bar graphs to facilitate the comments and clarifications offered by each participant during an online meeting. During this meeting, the panel openly discussed the statements/rationales on which agreement had not been reached. In some circumstances the discussion led the panelists to reconsider their initial opinions, whereas in others it resulted in the working group modifying the statements. A second round of blind online voting was then held. The results of the consensus process were tabulated and presented both descriptively and graphically. The process was closed on 16 June 2021.

### Questionnaire and consensus criteria

The group’s opinion on each statement and rationale was studied in terms of the score and the level of consensus reached by the panelists. The same criteria were applied to each of the statements and rationales. Opinions were expressed using a unique nine-point ordinal Likert-type scale, according to the model developed by UCLA-RAND Corporation (minimum score, 1 = full disagreement; maximum score, 9 = full agreement) [13]. This scale was split into three sections, indicating the level of agreement/disagreement: a score of 1–3 implicated rejection or disagreement (“not appropriate”); 4–6 implicated (“uncertainty”); and 7–9 implicated agreement/support (“appropriate”). Consensus was reached when (i) 75% or more of the respondents, i.e., at least 14 out of 18 experts (excluding the methodologists and the evidence review team), assigned a score within the 3-point ranges 1–3 or 7–9, which rejected or accepted the statements/rationales, respectively; and (ii) the median score also lay within these ranges [14]. The type of consensus achieved was determined by the median score: “agreement” was defined for a median score ≥ 7, and “disagreement” for a median score ≤ 3. A median score within the 4–6 range meant that most of the group had scored the items as “uncertain.”

## Results

**Fig. 1:**
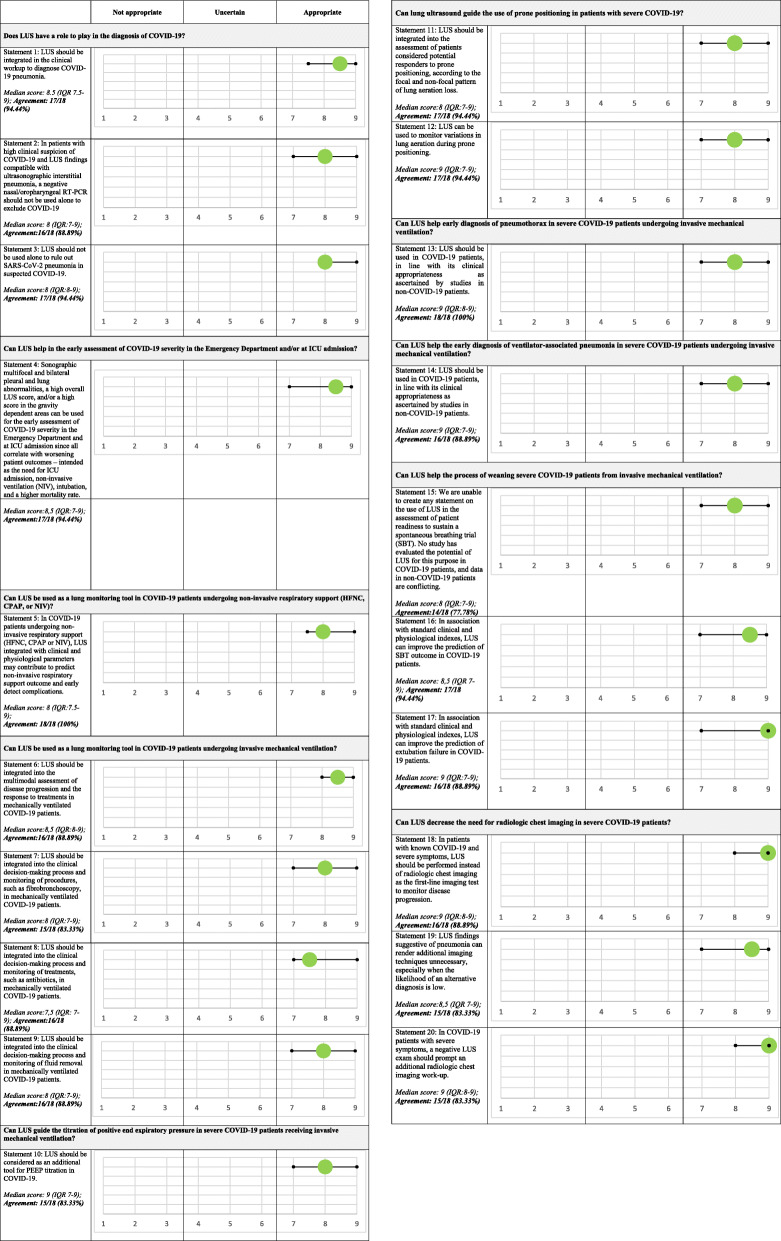
The median score (plus interquartile range) and agreement percentage for all the statements contained in the final consensus report

The panel was composed of a total of 18 experts, 4 methodologists, and 2 senior heads. The panel produced a total of 20 statements. The criteria for a consensus of agreement (i.e., a score in the range 7–9 provided by 75% or more of respondents, and a median score value also within this range) were met for 18 out of 20 statements in the first round of voting. Consensus was not achieved in relation to statements no. 5 and no. 17 (see Fig. S2, Additional file 2). After the second round of voting, consensus of agreement was achieved on all statements. The median score (plus interquartile range) and agreement percentage for all the statements contained in the final consensus report are shown in Fig. 1.

### Clinical questions and consensus-based statements

A) Does LUS have a role to play in the diagnosis of COVID-19?

- Statement 1: LUS should be integrated in the clinical workup to diagnose COVID-19 pneumonia.
- Statement 2: In patients with high clinical suspicion of COVID-19 and LUS findings compatible with ultrasonographic interstitial pneumonia, a negative nasal/oropharyngeal RT-PCR should not be used alone to exclude COVID-19.
- Statement 3: LUS should not be used alone to rule in and rule out SARS-CoV-2 pneumonia in suspected COVID-19.

Lung ultrasound has been shown to improve the diagnostic accuracy in patients who present with acute respiratory symptoms [15]. The reliance on the clinical presentation of patients is a highly ineffective approach for COVID-19 case identification. The sensitivity of using crackles on auscultation for the detection of parenchymal involvement in COVID-19 patients was just 8% when compared to CT as reference [11]. On the contrary, LUS performs better than standard tests for dyspnea in the Emergency Department [16, 17], permitting COVID-19 pneumonia to be diagnosed in patients with normal vital signs [11], and distinguishing between viral and bacterial pneumonias [17]. LUS is more accurate than chest X-ray in diagnosing respiratory conditions [18, 19], including interstitial diseases [20], pneumonia [21], and COVID-19 pneumonia [22]. If the pre-test probability of COVID-19 is low [23], a LUS bilateral A-pattern with sliding makes COVID-19 pneumonia unlikely owing to its high negative predictive value for pneumonia [24, 25]. The strong correlation between CT and LUS scans in COVID-19 patients supports the preferential use of LUS over CT in scenarios where CT is inappropriate (e.g., pregnancy) or difficult to obtain [26]. Real-time polymerase chain reaction (RT-PCR) testing is currently the standard diagnostic test for SARS-CoV-2 infection, and it is reported to have high specificity but only moderate sensitivity for diagnosing COVID-19 [27, 28]. Coupled with pre-test probability, bilateral B-lines, an irregular pleural line, and sub-pleural consolidations increase the likelihood of COVID-19 diagnosis [29, 30]. A recent meta-analysis of six observational studies and a case series describing a total of 122 symptomatic patients highlights the role of LUS in the diagnosis of COVID-19 [31]. Interstitial lung involvement, as depicted by the B-pattern, was the most common and consistent finding by LUS, and the presence of this finding in addition to other characteristic symptoms will increase the likelihood of diagnosis. The “light beam” artifact is a LUS sign that corresponds to the early appearance of “ground-glass” opacity on a CT scan, and it has been observed in most patients with COVID-19 pneumonia [32, 33]. Some authors have suggested the “light beam” sign to be specific to COVID-19 pneumonia, and its presence may increase the diagnostic likelihood in patients suspected of having COVID-19 [1]. However, LUS should always be integrated into a more comprehensive clinical evaluation, and LUS findings should be interpreted in light of the pre-test probability of COVID-19 pneumonia.

B) Can LUS help in the early assessment of COVID-19 severity in the Emergency Department and/or at ICU admission?

- Statement 4: Sonographic multifocal and bilateral pleural and lung abnormalities, a high overall LUS score, and/or a high score in the gravity dependent areas can be used for the early assessment of COVID-19 severity in the Emergency Department and at ICU admission since all correlate with worsening patient outcomes—intended as the need for ICU admission, non-invasive ventilation (NIV), intubation, and a higher mortality rate—although ICU admission still remains a clinical decision based on clinical assessment and some related exams.

**Fig. 2:**
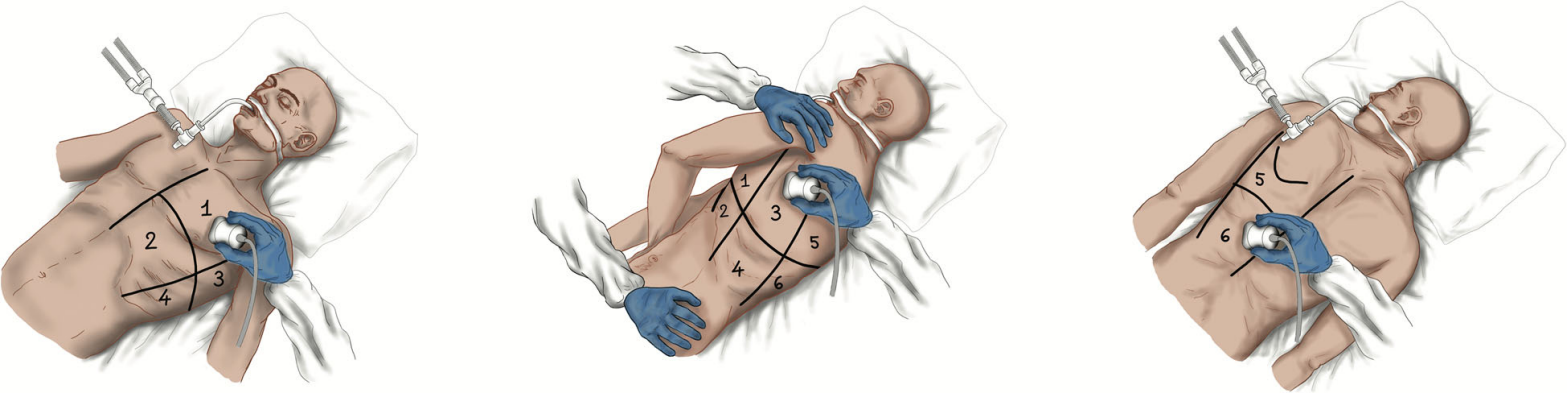
The lung ultrasound score (LUS) can be applied to assess the loss of aeration by dividing the thorax into 12 specific regions, six on the right and six on the left in supine or prone position assign each region a score from 0 (normal lung) to 3 (lung consolidation). Anterior, lateral, and posterior fields are identified by sternum, anterior, and posterior axillary lines. The entire examination can be performed without any change in patient’s position. Score: 0 = normal aeration (A-lines and lung sliding or maximum 2 well-spaced B-lines); score 1 = moderate loss of aeration (> = 3 well-spaced B-lines with lung sliding, coalescent B-lines/subpleural consolidations occupying < 50% of the pleural line); score 2 = severe loss of aeration (> = 3 well-spaced B-lines with lung sliding, coalescent B-lines/subpleural consolidations occupying clearly > 50% of the pleural line); score 3 = complete loss of aeration: lobar/hemilobar consolidation with predominant tissue like pattern

Several observational prospective cohort studies of COVID-19 patients have found a significant correlation between LUS findings and poor outcome, defined as ICU admission, increased likelihood of acute respiratory failure, the need for non-invasive or invasive mechanical ventilation, and a higher hospital mortality rate [34, 35]. In all these studies, LUS scores were based on a 4-level scoring system ranging from 0 to 3, but they differed in the number of areas scanned, which ranged from 6 to 14 lung regions [34–55]. Two groups of researchers analyzed the agreement between the different LUS score protocols and patient outcome in COVID-19 patients and concluded that, independently of the number of areas scanned, the accuracy in outcome prediction was higher when the protocol adopted included the evaluation of the gravity dependent areas [44, 45]. One case series and sixteen observational prospective cohort studies have provided consistent results suggesting that, independently of the protocol adopted, a moderate loss of aeration as assessed by LUS is associated with a higher need for NIV, whereas a severe loss of lung aeration is associated with an increased likelihood of ICU admission and mortality [34–41, 43–49, 51, 54]. In five observational prospective studies [40–42, 54, 55], an increased likelihood of NIV failure was associated with a severe loss of lung aeration as assessed by the LUS score, independently of the protocol adopted. In the light of this evidence, and independently of the number of areas scanned and the LUS score protocol adopted, the presence of sonographic multifocal and bilateral pleural and lung abnormalities together with a high overall LUS score and/or high scores in the gravity dependent areas may help in predicting NIV failure, the need for ICU admission, and hospital mortality in the emergency room. The 12-lung-region scoring system usually adopted in critically ill patients is described in Fig. 2.

C) Can LUS be used as a lung monitoring tool in COVID-19 patients undergoing non-invasive respiratory support (HFNC, CPAP, or NIV)?

- Statement 5: In COVID-19 patients undergoing non-invasive respiratory support (HFNC, CPAP, or NIV), LUS integrated with clinical and physiological parameters may contribute to predict non-invasive respiratory support outcome and early detect complications.

Although robust evidence in COVID-19 patients has yet to be gathered, apart from some interesting case series [56, 57] and a few observational cohort studies involving small sample sizes [58, 59], the role of LUS as a monitoring tool in the field of interstitial lung diseases has already been confirmed since the LUS score is known to correlate with lung aeration [60]. Based on all the evidence available in this setting [37, 39–41, 43, 49, 54, 56, 58], the daily assessment of the LUS score in COVID-19 patients undergoing non-invasive respiratory support can be considered an appropriate approach. A worsening overall LUS score, with or without a high LUS score in the gravity dependent areas, may be predictive of a worsening patient outcome and the failure of a 24-h non-invasive respiratory support trial [40–42, 54, 56]. On the other hand, a stable or decreasing overall LUS score after a 24-h non-invasive respiratory support trial, together with an improvement in the LUS score in the gravity dependent areas, may be associated with non-invasive respiratory support success. Furthermore, pneumothorax (PNX) and pneumomediastinum are common complications in severe COVID-19 respiratory failure [61, 62], even in patients undergoing non-invasive ventilation, especially if associated with exacerbating inspiratory efforts and high pleural pressure swings. The role of LUS in the early detection and monitoring of PNX is well-known. In fact, the sensitivity and specificity of LUS for the detection of even small occult PNX have been demonstrated to be high [63, 64]. In patients without subcutaneous emphysema, pneumomediastinum may be suspected once PNX has been ruled out in the presence of specific ultrasound findings in the subxiphoid view and/or in the anterolateral cervical region [65, 66].

D) Can LUS be used as a lung monitoring tool in COVID-19 patients undergoing invasive mechanical ventilation?

- Statement 6: LUS should be integrated into the multimodal assessment of disease progression and the response to treatments in mechanically ventilated COVID-19 patients.
- Statement 7: LUS should be integrated into the clinical decision-making process and monitoring of procedures, such as fibrobronchoscopy, in mechanically ventilated COVID-19 patients.
- Statement 8: LUS should be integrated into the clinical decision-making process and monitoring of treatments, such as antibiotics, in mechanically ventilated COVID-19 patients.
- Statement 9: LUS should be integrated into the clinical decision-making process and monitoring of fluid removal in mechanically ventilated COVID-19 patients.

Specific ultrasound findings provide useful information for the monitoring of mechanically ventilated patients [60, 67]. A complete examination requires only a few minutes and shows high inter-observer agreement even with ultra-portable devices [8–68]. Changes in the LUS score over time may help the assessment of disease progression and of the response to treatments in ARDS patients [69–72]. Monitoring the lung with LUS may improve the management of fluid balance in acute respiratory failure patients [73, 74]. The visualization of a newly appeared tissue-like pattern could point toward/be suggestive of reabsorption atelectasis (if a static or absent air-bronchogram is visualized) [75], ventilator-associated pneumonia (dynamic linear/arborescent air-bronchogram), or lung collapse with patent airways (dynamic punctiform air-bronchogram), thus indicating the need for dis-obstructive fibro-bronchoscopy, distal micro-biological sampling, or titration of positive pressure mechanical ventilation [76]. Pleural line assessment is also of interest in COVID-19 patients. The results of an in vitro study suggest that the persistence of subpleural consolidations and pleural irregularities may be a marker of fibro-proliferative diffuse alveolar damage [77]; in fact, these signs are observed in vivo at admission in patients with long-duration symptoms [78]. When combined with suggestive clinical parameters and compressive vein ultrasound, the presence of large subpleural consolidations are associated with a high probability of pulmonary embolism [79].

E) Can LUS guide the titration of positive end expiratory pressure in severe COVID-19 patients receiving invasive mechanical ventilation?

- Statement 10: LUS should be considered as an additional tool for PEEP titration in COVID-19.

The limitations of LUS as a tool for titrating PEEP levels in COVID-19 patients are the same as those applying to ARDS patients, namely, the limited capability to quantify recruitment and detect hyperinflation. PEEP-induced recruitment in COVID-19 is heterogeneous and limited in most patients [80]. LUS should be considered as an additional tool, complementary to respiratory mechanics and arterial blood gases, for determining and monitoring the effects of PEEP changes [81].

F) Can LUS guide the use of prone positioning in patients with severe COVID-19?

- Statement 11: LUS should be integrated into the assessment of patients considered potential responders to prone positioning, according to the focal and non-focal pattern of lung aeration loss.
- Statement 12: LUS can be used to monitor variations in lung aeration during prone positioning.

In conventional ARDS patients, lung ultrasound has been used to monitor the effects of prone positioning on lung reaeration [82, 83]. Haddam et al. showed that ARDS patients with a focal distribution of aeration loss as determined by LUS tended to experience greater reaeration during prone positioning, although it did not correlate with the oxygenation response [84]. In a second study, aeration changes in posterior fields induced by the first 3 h of prone positioning were significantly greater in patients with positive responses and associated with greater levels of oxygenation after 7 days of treatment. Moreover, the lung aeration changes correlated well with the reduction in dead space [85]. Rousset et al. confirm that prone responders present a greater LUS reaeration score at both an early and late stage of prone positioning, corresponding with an increase in end-expiratory lung volume [82]. Similarly, in COVID-19 pneumonia, the improvement in oxygenation following pronation was associated with an improvement in both the global and posterior LUS scores [85]. LUS was proven to be a reliable bedside tool for monitoring lung aeration across the different phases of prone positioning in ARDS patients undergoing invasive mechanical ventilation. Thus, lung aeration monitoring through LUS imaging during pronation could also constitute a valid solution for COVID-19 pneumonia, in which the early prediction of disease progression may prove to be fundamental for the delivery of appropriate healthcare.

G) Can LUS help early diagnosis of pneumothorax in severe COVID-19 patients undergoing invasive mechanical ventilation?

- Statement 13: LUS should be used in COVID-19 patients, in line with its clinical appropriateness as ascertained by studies in non-COVID-19 patients.

Analysis of the current literature failed to reveal the presence of studies specifically addressing LUS accuracy in pneumothorax diagnosis in COVID-19 patients. A single observational study by Li et al. [86], involving 42 patients, reported 4 cases of pneumothorax identified by LUS and two cases identified by chest X-ray, but no data on concordance were provided. A multi-center observational study by Zieleskiewicz et al. compared LUS performance against that of CT as the standard reference in the diagnosis of interstitial syndrome, consolidations, pleural effusion, pneumothorax, and pleural irregularity [5]. Unfortunately, no pneumothoraxes were present in 100 case samples; thus, the accuracy of LUS in pneumothorax diagnosis could not be estimated. Consistently, a low incidence of pneumothorax in COVID-19 patients was previously reported by Yang et al. in a single-center observational study of 52 patients [87]. Regarding pneumothorax diagnosis and monitoring, LUS applications in COVID-19 patients must currently rely on non-COVID-19 literature. To date, the role of LUS in pneumothorax diagnosis in the critically ill and in trauma patients is well-recognized [63]. Pneumothorax is defined by the presence of air in the pleural cavity, which, in the majority of cases, collects in the upper part (i.e., in the least dependent part), depending on patient position and habitus. Thus, where air collects, the visceral and parietal layers become separated, and a static pleural line replaces normal lung appearance due to the complete reflection of the US beam by the pleural air. This static pleural line has three features: the absence of lung sliding, the lack of B-lines, and the absence of lung pulse [88–90]. Pneumothorax can be ruled out in 100% of cases if lung sliding and B lines are present [7]. Only one sign is specific enough to rule pneumothorax in the lung-point [91]. This is the ultrasonographic sign that presents at the pneumothorax border where the visceral and parietal pleural contact each other again. However, lung-point can be challenging to find, or completely absent in very large pneumothorax cases. The accuracy of LUS in pneumothorax diagnosis has been evaluated in many studies, which have been synthesized into four meta-analyses published between 2011 and 2014 [92–95]. The pooled specificity of LUS is reported to vary between 98 and 99%, comparable to chest X-ray, while sensitivity is reported to be between 79 and 91%, far outperforming chest X-ray, the pooled sensitivity of which varies from 40 to 52%. Moreover, LUS has been shown to be a sensitive tool, with a low air volume threshold, able to detect even radio-occult pneumothoraxes (i.e., undetected by chest X-ray), which may inflate further during mechanical ventilation [96, 97]. LUS has also been shown to be a reliable means for semi-quantifying pneumothorax dimension, thus helping in treatment decision-making [98]. In the context of pneumothorax daily reassessment, some controversy exists regarding diagnostic consistency over time, with only one study reporting LUS to be adequately capable of detecting pneumothorax resolution after chest drain clamping and removal [99]. The presence of subcutaneous emphysema, which may accompany cases of pneumomediastinum, may limit this application, complicating mechanical ventilation in some COVID-19 patients [100].

H) Can LUS help the early diagnosis of ventilator-associated pneumonia in severe COVID-19 patients undergoing invasive mechanical ventilation?

- Statement 14: LUS should be used in COVID-19 patients, in line with its clinical appropriateness as ascertained by studies in non-COVID-19 patients.

Ventilator-associated pneumonia (VAP) is the most frequent nosocomial infection occurring in the ICU; it is associated with increased mortality, a greater use of antimicrobials, longer mechanical ventilation, and higher healthcare costs. VAP is suspected in patients under mechanical ventilation presenting fever/hypothermia, leukocytosis/leukopenia, purulent tracheal secretions, and impaired oxygenation [39]. No specific studies have been conducted so far to assess the accuracy of LUS in VAP diagnosis in COVID-19 patients. LUS is well suited to investigate interstitial and subpleural involvement in lung disease, and it is increasingly used in the ICU setting. The extent and severity of lung infiltrates can be described numerically with a reproducible and validated LUS score [39]. More recently, quantitative analysis of the LUS score has been proposed to make the interpretation of findings less operator dependent [6]. VAP-related injuries typically extend from the center to the periphery of the lung; when these lesions reach the subpleural regions, they become identifiable by LUS: a normal A-line pattern with lung sliding is replaced by focal areas of interstitial syndrome, represented by well-spaced B-lines, becoming progressively confluent into subpleural areas of consolidation, where air-bronchograms may be visualized [68]. LUS could become a tool for the detection of VAP in the ICU, but this application has only been investigated so far by three specific studies [6, 39, 68]. The current literature on LUS use in routine practice for COVID-19 patients is encouraging as it seems to accurately reflect disease progression [39]. Specific data on LUS accuracy for the diagnosis of VAP in severe COVID-19 patients undergoing invasive mechanical ventilation are still lacking, and well-designed studies are needed to validate this powerful tool [68].

I) Can LUS help the process of weaning severe COVID-19 patients from invasive mechanical ventilation?

- Statement 15: We are unable to create any statement on the use of LUS in the assessment of patient readiness to sustain a spontaneous breathing trial (SBT). No study has evaluated the potential of LUS for this purpose in COVID-19 patients, and data in non-COVID-19 patients are conflicting.
- Statement 16: In association with standard clinical and physiological indexes, LUS can improve the prediction of SBT outcome in COVID-19 patients.
- Statement 17: In association with standard clinical and physiological indexes, LUS can improve the prediction of extubation failure in COVID-19 patients.

The term weaning refers to the whole process leading to the liberation from mechanical ventilation and removal of the endotracheal tube [101]. The process includes a number of steps: (1) assessing spontaneous breathing trial (SBT) readiness; (2) conducting a SBT and assessing its outcome; and (3) predicting extubation failure, which would expose patients to an increased risk of death and prolonged ICU stay [101].

Readiness for SBT is commonly assessed by means of a composite evaluation, including clinical and physiological variables [101]. LUS imaging employed to ascertain patient readiness to undergo an SBT has produced conflicting results in non-COVID-19 patients. In critically ill neurosurgical patients, a high LUS score prior to a 1-h SBT conducted using a T-tube was associated with a higher risk of SBT failure [102]. Conversely, data from a bi-center, prospective, observational investigation, carried out in a heterogeneous population of 250 ready-to-wean patients, showed that B-line predominance prior to sustaining a T-tube SBT lasting 30–120 min was a very weak predictor of SBT outcome, with 47% sensitivity, 64% specificity, a positive predictive value of 25%, and a negative predictive value of 82% [103]. The authors concluded that the presence of B-lines on a simplified 4-zone LUS protocol should not interfere with the decision to initiate weaning procedures [103].

The outcomes of SBTs and extubation depend on a variety of factors, such as cardiovascular dysfunction, inability of the respiratory muscles to sustain an excessive work of breathing, neuromuscular disorders, severe anemia, altered metabolic, nutritional, or neuropsychological conditions, and the inability to clear secretions [101]. While LUS is not helpful for evaluating some of these factors, it may add to the physician’s decision-making when associated with clinical and physiological variables and in some cases with cardiac and diaphragm ultrasound assessment [104–106]. In critically ill patients, following the reduction of ventilatory support, the extension of regions affected by aeration loss and pulmonary edema may contribute to weaning failure [105] and can be identified and quantified by LUS [107, 108]. Indeed, variations in LUS patterns reflect changes in pulmonary aeration, which is the final result of different pathways [108–110]. Patients who fail an SBT show lung de-recruitment and inhomogeneity according to electric impedance tomography analysis [111]. In patients invasively ventilated for more than 48 h, a 1-h SBT conducted using a T-tube showed a LUS greater than that observed in patients with successful SBT [104]. These results were subsequently confirmed in a second study conducted in the same setting [109]. In patients intubated for acute respiratory failure of different etiologies undergoing an SBT either in T-tube mode or with 8 cmH_2_O of inspiratory pressure support and 5 cmH_2_O of positive end-expiratory pressure, the discriminative power of LUS for successful weaning was 0.8 with a sensitivity and specificity of 0.76 and 0.73, respectively [112]. An increase in B-lines to ≥ 6 on four anterior points during a 60-min SBT in T-tube mode was shown to predict weaning-induced pulmonary edema with a sensitivity of 88% (64–98) and a specificity of 88% (62–98) [110]. In elderly patients undergoing an SBT, global and anterolateral LUS predicted failure with a discriminative power of 0.80 and 0.79, respectively [108].

Combining LUS with cardiac and diaphragmatic ultrasound assessment has the potential to improve weaning failure prediction, providing insights into the origin of reduced pulmonary aeration [113, 114]. A modified LUS assessment and diaphragmatic thickening assessment were associated with high predictive accuracy of successful extubation, with an AUC of 0.78 and 0.76, respectively, which increased up to 0.83 when considering both assessments together [112]; however, the relationship between LUS and diaphragm thickening during an SBT seems to vary according to the degree of pulmonary aeration [115], as well as to the considered subpopulation [116]. LUS in association with the brain natriuretic peptide test, diaphragm dysfunction assessment, and left atrial pressure measurement is a better predictor of weaning failure than LUS alone (AUC 0.91 vs 0.76) [117].

In non-COVID-19 patients, LUS was successfully applied to predict extubation outcome. Regardless of the primary cause of the weaning failure, LUS performed in 100 ICU patients during successful SBTs in T-tube mode was able to predict the occurrence of post-extubation distress [112]. A LUS score higher than 17 was associated with an increased risk of extubation failure, whereas a gray zone was identified for scores ranging from 13 to 17. In patients who successfully passed the SBT with a LUS score > 13, non-invasive ventilation was proposed as a rescue strategy to prevent re-intubation [104]. LUS assessment during successful SBTs with support pressure ventilation < 7 cmH2O and no PEEP revealed the occurrence of multiple B-lines as a predictor of post-extubation distress within the first 48 h after extubation [113]. Despite the lack of data in COVID-19 patients, the pathophysiological insights gained from LUS during a SBT to predict extubation failure might be translated to COVID-19 patients. This suggestion arrives from the consideration that observational studies have evaluated the reliability of LUS as a monitoring tool in COVID-19 patients, despite it being a different clinical setting to weaning [42].

Changes in the LUS score during SBTs in COVID-19 patients could add some information concerning the loss of aeration. Cardiac and diaphragm ultrasound assessments might also be able to enhance SBT outcome prediction.

L) Can LUS decrease the need for radiologic chest imaging in severe COVID-19 patients?

*Statement 18: In patients with known COVID-19 and severe symptoms, LUS should be performed instead of radiologic chest imaging as the first-line imaging test to monitor disease progression.*

- Statement 19: LUS findings suggestive of pneumonia can render additional imaging techniques unnecessary, especially when the likelihood of an alternative diagnosis is low.
- Statement 20: In COVID-19 patients with severe symptoms, a negative LUS exam should prompt an additional radiologic chest imaging work-up.

Monitoring critically ill patients by serial chest X-ray is not advisable due to the technique’s low sensitivity and long execution time; moreover, it represents a suboptimal allocation of available resources [19].

Conversely, LUS demonstrates high agreement with chest computer tomography (CT), closely mirrors the longitudinal changes found by CT without exposing the personnel to infection risks, and is quick to perform [9]. If integrated into the daily routine examinations, LUS results appear to accurately reflect disease progression, thus reducing the need for chest X-ray and CT [41–118]. Moreover, LUS has been shown capable of monitoring the evolution of severe COVID-19 pneumonia after hospital discharge, supporting its integration into clinical predictive models of residual lung injury, thus providing an alternative imaging modality for the diagnosis and monitoring of critically ill COVID-19 patients [119].

If a patient presents LUS findings suggestive of pneumonia together with a low pre-test probability of an alternative or secondary diagnosis, an additional imaging modality may not be necessary [1–120]; however, a chest CT on admission performs better than LUS for COVID-19 diagnosis, at varying disease prevalence [38], and LUS is highly sensitive but not specific for COVID-19 [121]. Thus, the presence of pre-existing conditions, such as chronic obstructive pulmonary disease or heart failure, and the prevalence of venous thromboembolism in patients with COVID-19 must be taken into account when deciding whether to pursue or not additional chest imaging modalities, which should be individualized on a patient-by-patient basis [120].

In patients with severe symptoms, the probability of entirely normal radiologic findings is low. Thus, in patients with severe symptoms, a negative LUS should prompt additional radiologic chest imaging work-up by chest CT owing to its higher accuracy in imaging centrally based abnormalities and diagnosis of pulmonary embolism [122–124].

## Discussion

The panel produced 20 statements in relation to 10 clinical questions on the bedside use of LUS in COVID-19 critically ill patients, summarizing the latest available literature and the direct experience of the expert panelists. As the use of LUS became ineluctable during this pandemic, anesthesiologists and intensive care physicians have been quick to incorporate this important tool into their armamentarium [1, 2, 4–6, 9]. This also reinforces the need to impose training and certification on the use of this tool within our discipline in order to ensure its wider implementation in the near future [125]. Two other groups have proposed consensuses on the use of LUS in the setting of COVID-19 [12, 126]. The Canadian consensus statement on the use of LUS was focused on the assessment of medical inpatients to confirm or rule out the diagnosis of COVID-19 pneumonia [12]. Our consensus not only confirms the use of LUS for COVID-19 pneumonia diagnosis but also details its use as a monitoring tool in the critically ill. A second multi-organ point-of-care ultrasound consensus for COVID-19 patients included a whole-body ultrasound approach [126]. While it supports the use of LUS as an accurate diagnostic tool, the specific use of LUS in routine ICU work was not detailed. Two other society guidelines have been produced: the first by the German societies of clinical acute, emergency, and intensive care medicine and radiology [127] and the second by the British Medical Ultrasound Society (BMUS) [128]. Both are mainly focused on imaging and monitoring techniques in COVID-19, but do not detail the use of LUS in the critically ill. Finally, the European Society of Radiology recently highlighted the role of lung ultrasound in COVID-19 disease in the ICU, supporting its use “to track the evolution of disease during follow-up and to monitor lung recruitment maneuvers, the response to prone position ventilation and the controlling of extracorporeal membrane therapy” [2].

## Conclusions

In conclusion, our expert consensus is the first detailing the use of LUS for diagnosis, management, and monitoring of COVID-19 pneumonia in the critically ill patient. We hope that the statements produced will help ICU physicians in their daily clinical practice while facing the ongoing pandemic.

## Supplementary Information

**Additional file 1.** Search strategy

**Additional file 2: Table S1.** PICO question and criteria for inclusion/exclusion of articles. **Fig. S1.** PRISMA Flow Diagram. **Fig. S2.** Report of the first round of voting. For each statement, median score, interquartile range and proportion (percentage) of agreement are reported. Green circle (or red) represents the median score while the horizontal bar represents the interquartile-range. CPAP: Continuous positive airway pressure; COVID-19: Coronavirus diseases 2019; LUS: Lung ultrasound; HFNC: high flow nasal canula; IQR: interquartile range; ICU: Intensive care unit; NIRS: Noninvasive respiratory support; NIV: Noninvasive ventilation; PEEP: Positive end expiratory pressure; RT-PCR: Real-time polymerase chain reaction; SBT: Spontaneous breathing trial

**Additional file 3.** Full output of the search and the selection of potentially relevant articles

## Data Availability

The data of the present expert consensus is available, on reasonable request, by contacting the corresponding author.

## Abbreviations

LUS: Lung ultrasound
COVID-19: Coronavirus disease 2019
SARS-CoV2: Severe acute respiratory syndrome coronavirus 2
ARDS: Acute respiratory distress syndrome
CT: Computed tomography
PEEP: Positive end-expiration pressure
ICU: Intensive care unit
HFNC: High flow nasal cannula
NIV: Non-invasive ventilation
CPAP: Continuous positive airway pressure
PNX: Pneumothorax
VAP: Ventilator-associated pneumonia
SBT: Spontaneous breathing trial
BMUS: British Medical Ultrasound Society
