# Supplementary material for: Italian Society of Anesthesia, Analgesia, Resuscitation, and Intensive Care expert consensus statement on the use of lung ultrasound in critically ill patients with coronavirus disease 2019 (ITACO)
Source: J Anesth Analg Crit Care. 2021 Nov 24;1:16. doi: 10.1186/s44158-021-00015-6 (PMC8611396; doi:10.1186/s44158-021-00015-6)
Supplement: Supplementary file 1 — Additional file 1. Search strategy [file 44158_2021_15_MOESM1_ESM.docx]

**Online Resource 1**

ITAlian Society of AnesthEsia, Analgesia, RESUSCITATION and Intensive Care Expert Consensus Statement on the Use of Lung Ultrasound in Critical ILL Patients with COronavirus Disease 2019 (ITACO)

Francesco Mojoli^1^, Luigi Vetrugno^2,3*^, Andrea Cortegiani^4,5^, Elena Giovanna Bignami^6^, Mariachiara Ippolito^4,5^, Daniele Orso^2^, Francesco Corradi^7,8^, Gianmaria Cammarota^9^, Silvia Mongodi^1^, Enrico Boero^10^, Carmine Iacovazzo^11^, Maria Vargas^11^, Daniele Poole^12^, Daniele Guerino Biasucci^13^, Paolo Persona^14^, Tiziana Bove^2,3^, Lorenzo Ball^15,16^, Davide Chiumello^17^, Francesco Forfori^7^, Edoardo De Robertis^9^, Paolo Pelosi^15,16^, Paolo Navalesi^14^, Antonino Giarratano^4,5^ and Flavia Petrini^18^

^1^Department of Clinical-Surgical, Diagnostic, and Pediatric Sciences, Unit of Anesthesia and Intensive Care, University of Pavia, Pavia, Italy

^2^Department of Medicine, University of Udine, Udine, Italy

^3^University-Hospital of Friuli Centrale, ASU FC, Udine, Italy

^4^Department of Surgical, Oncological and Oral Science (Di.Chir.On.S), University of Palermo, Palermo, Italy

^5^Department of Anesthesia Intensive Care and Emergency, Policlinico Paolo Giaccone, Palermo, Italy

^6^Anesthesiology, Critical Care and Pain Medicine Division, Department of Medicine and Surgery, University of Parma, Parma, Italy

^7^Department of Surgical, Medical and Molecular Pathology and Critical Care Medicine, University of Pisa, Pisa, Italy

^8^Department of Anesthesia and Intensive Care, “Ente Ospedaliero Ospedali Galliera”, Genova, Italy

^9^Department of Medicine and Surgery, University of Perugia, Perugia, Italy

^10^Anesthesia and Intensive Care Unit, San Giovanni Bosco Hospital, Turin, Italy

^11^Department of Neurosciences, Reproductive and Odontostomatological Sciences, University of Naples "Federico II", Naples, Italy

^12^Anesthesia and Intensive Care Operative Unit, S. Martino Hospital, Belluno, Italy

^13^Department of Anesthesia and Intensive Care, Fondazione Policlinico Universitario “A. Gemelli,” Rome, Italy

^14^ UOC Anesthesia and Intensive Care Unit, University Hospital of Padua, Padua, Italy

^15^ Department of Surgical Sciences and Integrated Diagnostics (DISC), University of Genoa, Genoa, Italy

^16^Anesthesia and Critical Care, San Martino Policlinico Hospital, IRCCS for Oncology and Neurosciences, Genoa, Italy

^17^Department of Anesthesia and Intensive Care, ASST Santi Paolo e Carlo, San Paolo University Hospital, Milan, Milan, Italy

^18^President Italian Society of Anesthesia, Analgesia, Resuscitation, and Intensive Care (SIAARTI), Rome, Italy

***Corresponding author:**

Luigi Vetrugno, MD, Associate Professor

Department of Medicine, University of Udine, Udine Italy

33100, Via Colugna n 50, Udine, Italy

Phone: +39 0432 559501- Fax: +39 0432 559502

e-mail: [luigi.vetrugno@uniud.it](mailto:luigi.vetrugno@uniud.it)

ORCID: <https://orcid.org/0000-0003-3745-8368>

**Search strategy**

**PubMed**

Search: **((COVID-19) OR (sars-cov-2) OR (coronavirus) OR (covid)) AND ((ultrasound) OR (echography) OR (echo) OR (LUS))**

("covid 19"[All Fields] OR "covid 19"[MeSH Terms] OR "covid 19 vaccines"[All Fields] OR "covid 19 vaccines"[MeSH Terms] OR "covid 19 serotherapy"[All Fields] OR "covid 19 serotherapy"[Supplementary Concept] OR "covid 19 nucleic acid testing"[All Fields] OR "covid 19 nucleic acid testing"[MeSH Terms] OR "covid 19 serological testing"[All Fields] OR "covid 19 serological testing"[MeSH Terms] OR "covid 19 testing"[All Fields] OR "covid 19 testing"[MeSH Terms] OR "sars cov 2"[All Fields] OR "sars cov 2"[MeSH Terms] OR "severe acute respiratory syndrome coronavirus 2"[All Fields] OR "ncov"[All Fields] OR "2019 ncov"[All Fields] OR (("coronavirus"[MeSH Terms] OR "coronavirus"[All Fields] OR "cov"[All Fields])) OR ("sars cov 2"[MeSH Terms] OR "sars cov 2"[All Fields] OR "sars cov 2"[All Fields]) OR ("coronavirus"[MeSH Terms] OR "coronavirus"[All Fields] OR "coronaviruses"[All Fields]) OR ("sars cov 2"[MeSH Terms] OR "sars cov 2"[All Fields] OR "covid"[All Fields] OR "covid 19"[MeSH Terms] OR "covid 19"[All Fields])) AND ("diagnostic imaging"[MeSH Subheading] OR ("diagnostic"[All Fields] AND "imaging"[All Fields]) OR "diagnostic imaging"[All Fields] OR "ultrasound"[All Fields] OR "ultrasonography"[MeSH Terms] OR "ultrasonography"[All Fields] OR "ultrasonics"[MeSH Terms] OR "ultrasonics"[All Fields] OR "ultrasounds"[All Fields] OR "ultrasound s"[All Fields] OR ("diagnostic imaging"[MeSH Subheading] OR ("diagnostic"[All Fields] AND "imaging"[All Fields]) OR "diagnostic imaging"[All Fields] OR "echography"[All Fields] OR "ultrasonography"[MeSH Terms] OR "ultrasonography"[All Fields] OR "echographies"[All Fields]) OR ("echo"[Journal] OR "echo"[All Fields]) OR "LUS"[All Fields])

**Translations**

**COVID-19:** ("COVID-19" OR "COVID-19"[MeSH Terms] OR "COVID-19 Vaccines" OR "COVID-19 Vaccines"[MeSH Terms] OR "COVID-19 serotherapy" OR "COVID-19 serotherapy"[Supplementary Concept] OR "COVID-19 Nucleic Acid Testing" OR "covid-19 nucleic acid testing"[MeSH Terms] OR "COVID-19 Serological Testing" OR "covid-19 serological testing"[MeSH Terms] OR "COVID-19 Testing" OR "covid-19 testing"[MeSH Terms] OR "SARS-CoV-2" OR "sars-cov-2"[MeSH Terms] OR "Severe Acute Respiratory Syndrome Coronavirus 2" OR "NCOV" OR "2019 NCOV" OR (("coronavirus"[MeSH Terms] OR "coronavirus" OR "COV"))

**sars-cov-2:** "sars-cov-2"[MeSH Terms] OR "sars-cov-2"[All Fields] OR "sars cov 2"[All Fields]

**coronavirus:** "coronavirus"[MeSH Terms] OR "coronavirus"[All Fields] OR "coronaviruses"[All Fields]

**covid:** "sars-cov-2"[MeSH Terms] OR "sars-cov-2"[All Fields] OR "covid"[All Fields] OR "covid-19"[MeSH Terms] OR "covid-19"[All Fields]

**ultrasound:** "diagnostic imaging"[Subheading] OR ("diagnostic"[All Fields] AND "imaging"[All Fields]) OR "diagnostic imaging"[All Fields] OR "ultrasound"[All Fields] OR "ultrasonography"[MeSH Terms] OR "ultrasonography"[All Fields] OR "ultrasonics"[MeSH Terms] OR "ultrasonics"[All Fields] OR "ultrasounds"[All Fields] OR "ultrasound's"[All Fields]

**echography:** "diagnostic imaging"[Subheading] OR ("diagnostic"[All Fields] AND "imaging"[All Fields]) OR "diagnostic imaging"[All Fields] OR "echography"[All Fields] OR "ultrasonography"[MeSH Terms] OR "ultrasonography"[All Fields] OR "echographies"[All Fields]

**echo:** "echo"[All Fields]

**EMBASE**

('covid 19'/exp OR 'covid 19' OR 'sars cov 2'/exp OR 'sars cov 2' OR 'coronavirus'/exp OR coronavirus OR covid) AND ('ultrasound'/exp OR ultrasound OR 'echography'/exp OR echography OR 'echo'/exp OR echo OR lus)

**medRxiv and bioRxiv**

'covid 19' OR 'sars cov 2' OR coronavirus OR covid) AND (ultrasound OR echography OR echo OR lus)
