# Supplementary material for: Italian Society of Anesthesia, Analgesia, Resuscitation, and Intensive Care expert consensus statement on the use of lung ultrasound in critically ill patients with coronavirus disease 2019 (ITACO)
Source: J Anesth Analg Crit Care. 2021 Nov 24;1:16. doi: 10.1186/s44158-021-00015-6 (PMC8611396; doi:10.1186/s44158-021-00015-6)
Supplement: Supplementary file 2 — Additional file 2: Table S1. PICO question and criteria for inclusion/exclusion of articles. Fig. S1. PRISMA Flow Diagram. Fig. S2. Report of the first round of voting. For each statement, median score, interquartile range and proportion (percentage) of agreement are reported. Green circle (or red) represents the median score while the horizontal bar represents the interquartile-range. CPAP: Continuous positive airway pressure; COVID-19: Coronavirus diseases 2019; LUS: Lung ultrasound; HFNC: high flow nasal canula; IQR: interquartile range; ICU: Intensive care unit; NIRS: Noninvasive respiratory support; NIV: Noninvasive ventilation; PEEP: Positive end expiratory pressure; RT-PCR: Real-time polymerase chain reaction; SBT: Spontaneous breathing trial [file 44158_2021_15_MOESM2_ESM.docx]

**Online Resource 2**

ITAlian Society of AnesthEsia, Analgesia, RESUSCITATION and Intensive Care Expert Consensus Statement on the Use of Lung Ultrasound in Critical ILL Patients with COronavirus Disease 2019 (ITACO)

Francesco Mojoli^1^, Luigi Vetrugno^2,3*^, Andrea Cortegiani^4,5^, Elena Giovanna Bignami^6^, Mariachiara Ippolito^4,5^, Daniele Orso^2^, Francesco Corradi^7,8^, Gianmaria Cammarota^9^, Silvia Mongodi^1^, Enrico Boero^10^, Carmine Iacovazzo^11^, Maria Vargas^11^, Daniele Poole^12^, Daniele Guerino Biasucci^13^, Paolo Persona^14^, Tiziana Bove^2,3^, Lorenzo Ball^15,16^, Davide Chiumello^17^, Francesco Forfori^7^, Edoardo De Robertis^9^, Paolo Pelosi^15,16^, Paolo Navalesi^14^, Antonino Giarratano^4,5^ and Flavia Petrini^18^

^1^Department of Clinical-Surgical, Diagnostic, and Pediatric Sciences, Unit of Anesthesia and Intensive Care, University of Pavia, Pavia, Italy

^2^Department of Medicine, University of Udine, Udine, Italy

^3^University-Hospital of Friuli Centrale, ASU FC, Udine, Italy

^4^Department of Surgical, Oncological and Oral Science (Di.Chir.On.S), University of Palermo, Palermo, Italy

^5^Department of Anesthesia Intensive Care and Emergency, Policlinico Paolo Giaccone, Palermo, Italy

^6^Anesthesiology, Critical Care and Pain Medicine Division, Department of Medicine and Surgery, University of Parma, Parma, Italy

^7^Department of Surgical, Medical and Molecular Pathology and Critical Care Medicine, University of Pisa, Pisa, Italy

^8^Department of Anesthesia and Intensive Care, “Ente Ospedaliero Ospedali Galliera”, Genova, Italy

^9^Department of Medicine and Surgery, University of Perugia, Perugia, Italy

^10^Anesthesia and Intensive Care Unit, San Giovanni Bosco Hospital, Turin, Italy

^11^Department of Neurosciences, Reproductive and Odontostomatological Sciences, University of Naples "Federico II", Naples, Italy

^12^Anesthesia and Intensive Care Operative Unit, S. Martino Hospital, Belluno, Italy

^13^Department of Anesthesia and Intensive Care, Fondazione Policlinico Universitario “A. Gemelli,” Rome, Italy

^14^ UOC Anesthesia and Intensive Care Unit, University Hospital of Padua, Padua, Italy

^15^ Department of Surgical Sciences and Integrated Diagnostics (DISC), University of Genoa, Genoa, Italy

^16^Anesthesia and Critical Care, San Martino Policlinico Hospital, IRCCS for Oncology and Neurosciences, Genoa, Italy

^17^Department of Anesthesia and Intensive Care, ASST Santi Paolo e Carlo, San Paolo University Hospital, Milan, Milan, Italy

^18^President Italian Society of Anesthesia, Analgesia, Resuscitation, and Intensive Care (SIAARTI), Rome, Italy

***Corresponding author:**

Luigi Vetrugno, MD, Associate Professor

Department of Medicine, University of Udine, Udine Italy

33100, Via Colugna n 50, Udine, Italy

Phone: +39 0432 559501- Fax: +39 0432 559502

e-mail: [luigi.vetrugno@uniud.it](mailto:luigi.vetrugno@uniud.it)

ORCID: <https://orcid.org/0000-0003-3745-8368>

Table S1. PICO question and criteria for inclusion/exclusion of articles.

| PICO QUESTION |
| --- |
| P: Critically ill patients with acute respiratory failure due to proven or suspected infection by SARS-CoV-2 |
| I: Lung ultrasound |
| C: None |
| **O:** Related to diagnosis, prognosis or treatment selection according to the clinical questions of the consensus |
| **INCLUSION CRITERIA** |
| - Randomized or non randomized studies (both prospective or retrospective observational) |
| - Case series reporting data on more than 10 relevant patients |
| - Systematic reviews, with or without meta-anallysis |
| - Expert consensus |
| - Guidelines |
| **EXCLUSION CRITERIA** |
| - Case reports |
| - Narrative reviews |
| - Abstract |
| - Article not in English |

Fig. S1 PRISMA Flow Diagram


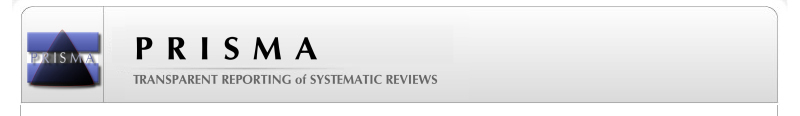
**PRISMA 2009 Flow Diagram**

Studies included in qualitative synthesis
(n =111)

Full-text articles excluded, with reasons
(n =38)

9 Case reports or case series with less than ten patients

6 Abstracts/Conference proceedings

6 Non-English language

6 Narrative review

4 Full-text unavailable/Articles not found

3 Retracted articles or notices of retraction/expression of concern

3 No data of interest

1 Commentary, no data

Full-text articles assessed for eligibility
(n =149)

Records identified through database searching
(n =6450)

EMBASE, n=2351

PubMed, n=3872

medRxiv, n=198

bioRxiv, n=29

Additional records identified through other sources
(n =3)

Records screened
(n =6453)

Records excluded
(n = 6304)

## Identification

## Eligibility

## Included

## Screening

Fig. S2. Report of the first round of voting. For each statement, median score, interquartile range and proportion (percentage) of agreement are reported. Green circle (or red) represents the median score while the horizontal bar represents the interquartile-range.

CPAP: Continuous positive airway pressure; COVID-19: Coronavirus diseases 2019; LUS: Lung ultrasound; HFNC: high flow nasal canula; IQR: interquartile range; ICU: Intensive care unit; NIRS: Noninvasive respiratory support; NIV: Noninvasive ventilation; PEEP: Positive end expiratory pressure; RT-PCR: Real-time polymerase chain reaction; SBT: Spontaneous breathing trial

**
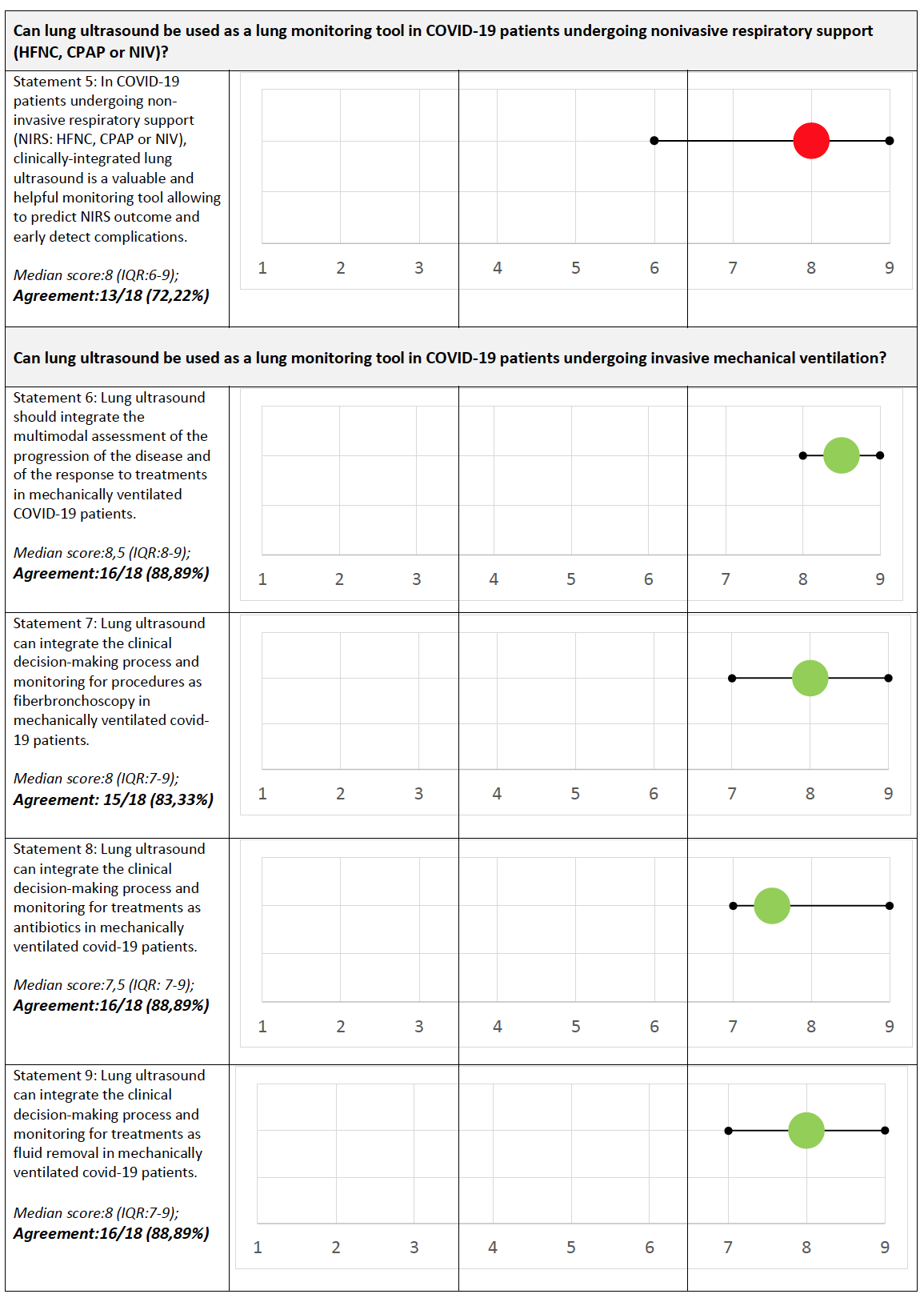
**

**
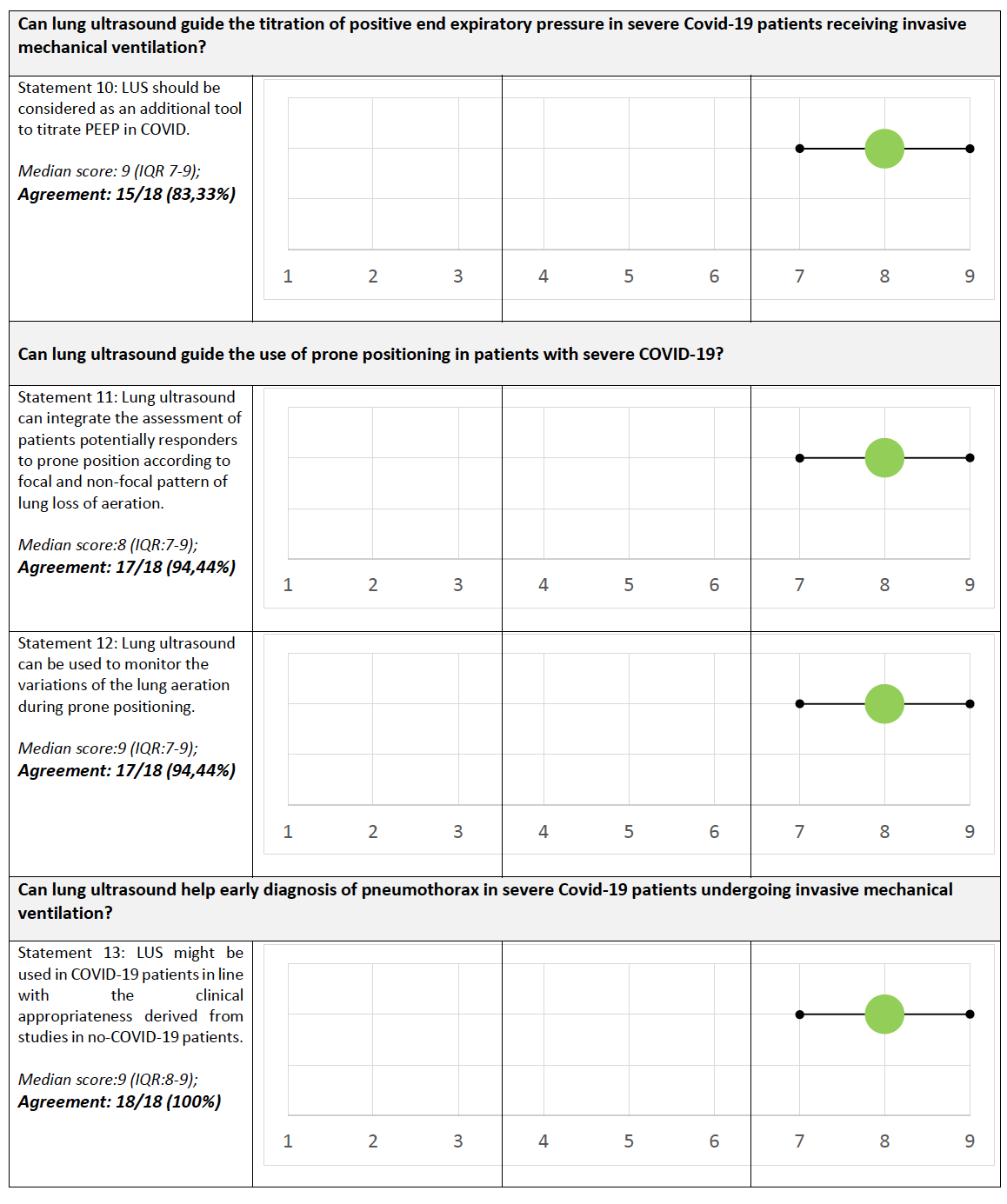
**

**
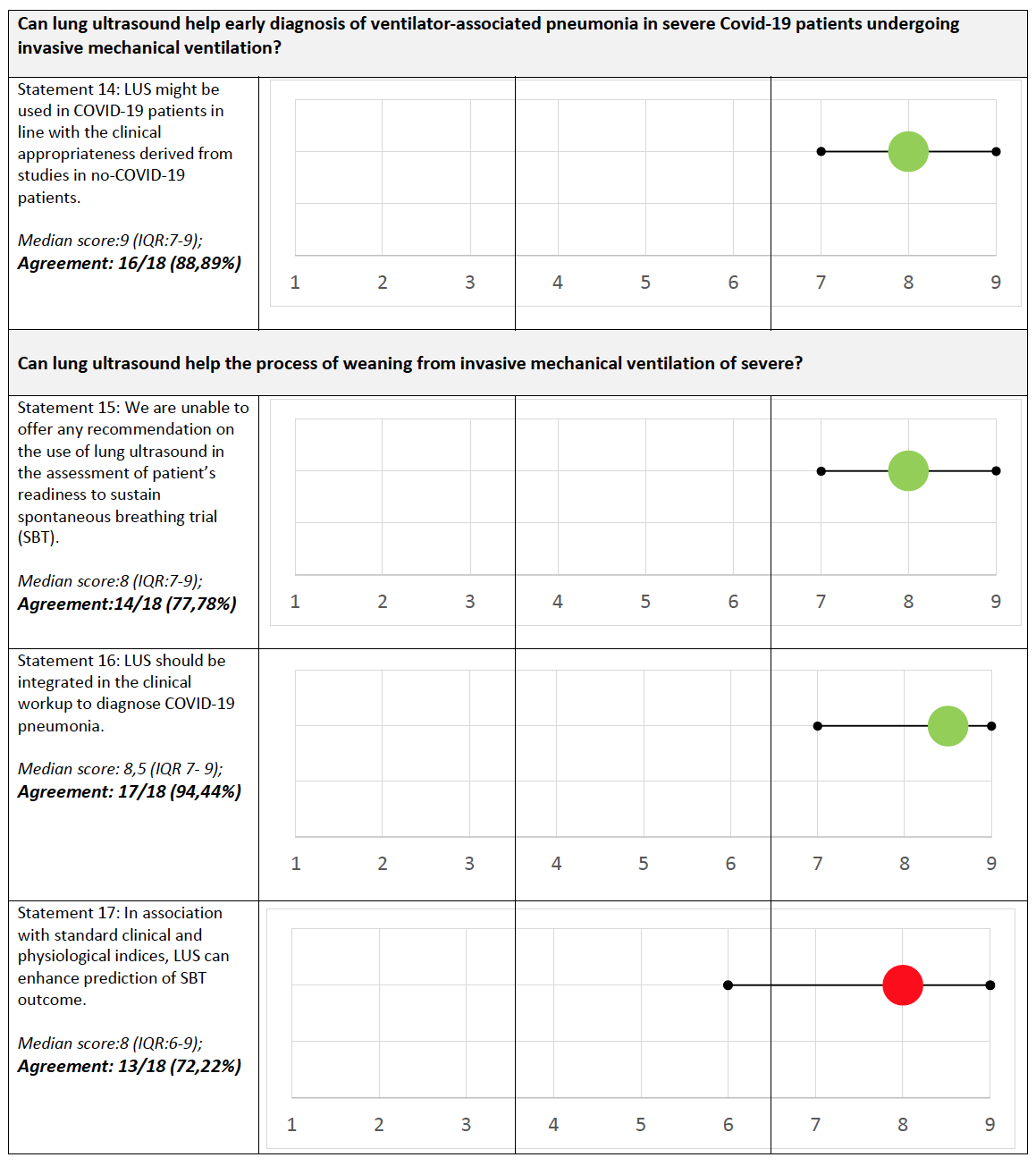
**

**
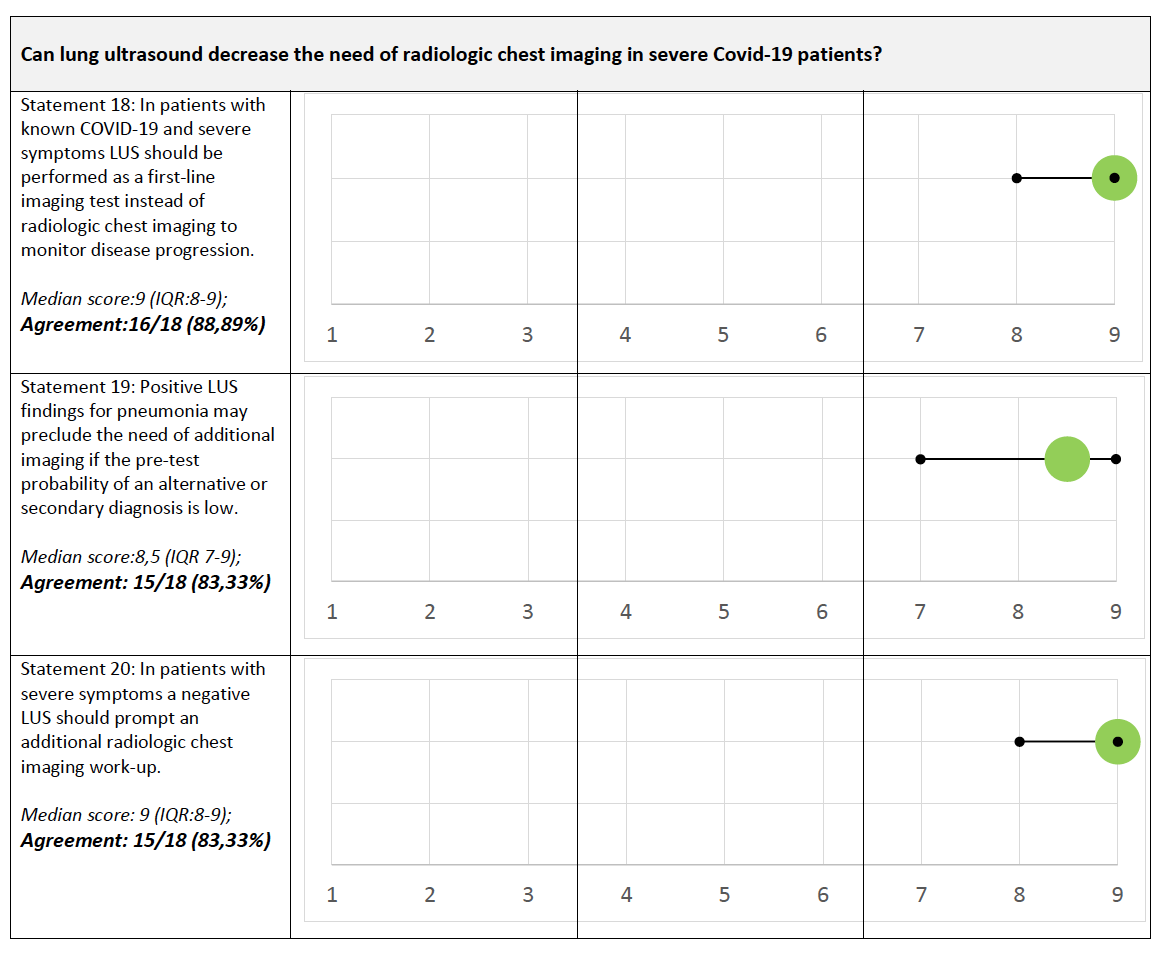
**
